# The Citizen of the Future

**Published:** 1924-08

**Authors:** 


### THE CITIZEN OF THE FUTURE.

### CONFERENCE ON INFANT WELFARE.

The Third English-Speaking Conference on Infant Welfare opened on July 1st, when Caxton Hall was crowded to its utmost capacity with delegates from thirty different countries. The social side of the Conference began the evening before with a reception at the Forum Club. A forcible presidential address was delivered by the Minister of Health, who laid particular stress on the human side of health work, pointing out that the ability to cultivate friendly relations was one of its most important factors. Their desire was not, he said, to relieve the mother of her functions and duties, but to enable her to perform them satisfactorily. Referring to the statistics of maternal mortality, he urged that motherhood should be made less hazardous, and he was about to issue a circular to local authorities asking them if they can suggest means by which child-birth mortality may be lowered. Care of the expectant mother was as essential as skilled attention and proper solicitude during confinement, but though institutions were at present necessary they could not be content until people possessed homes in which it was safe for babies to be born. Amid enthusiasm Mr. Wheatley said that his Government considered it false economy to starve facilities for bringing into the world healthy British babies, and he hoped that the period of economy had not damped enthusiasm. The Ministry was prepared to make grants to all well-considered schemes proposed by local authorities.

### Training of the Health Worker.

On the same morning an interesting address was given by Dr. Janet Lane-Claypon on "The Training of the Health Worker." Her education must remain debatable until the work she has to do has been in some degree settled. How far could curative and preventive medicine be satisfactorily interwoven in the work of the health visitor? Dr. Lane-Claypon thinks that the health worker must be mainly a preventive worker. "She cannot feel the glow of satisfaction of the curative worker because she can never really know that by remedying such states she prevented illness in any particular child."

### Neither Nurse nor Midwife.

Curative work should be undertaken by the nursing services. The midwife has her recognised sphere, and the health worker should be responsible under due supervision for the child till it leaves school, if possible. If the health worker is to appreciate the effects of defective public or personal hygiene she must have some experience of sickness, and for this she requires some hospital training. She must also have an elementary acquaintance with midwifery. But she cannot spare time for and does not require the full training of a hospital nurse as given in this country, nor that of a midwife. The health worker needed training in sanitation in its widest sense, in the normal working of the body at different ages of life, in the elements of social economics, and in local government or civics, as it is often termed. She should be a specialised worker, with a special training. She would neither be able nor be entitled to practise as a fully trained sick nurse or as a midwife. This implies that there would be a profession of "Health Worker," which would require due recognition, with adequate salaries and prospects of advancement just as there are in any other definite profession. In the afternoon the Conference discussed "The Unmarried Mother and her Child," and on the following morning "Child Adoption" and "Overseas Settlements for Children" were debated.

### Day Nursery Work.

On Wednesday afternoon the Hon. Mrs. Eustace Hills presided, when Sir Bruce Bruce-Porter read a paper on "The Preventive Side of Day Nursery Work." Most of the ailments which cripple and kill in middle age, he said, have their roots in childhood. Faults in breathing which result in adenoids and enlarged tonsils, defective eyesight, inherited venereal disease, the significance of constant bilious attacks, often in his opinion the root-cause of appendicitis in later life, all these are early recognised in the Day Nursery, where a child is under skilled observation. It is possible there to induce the mother to dress her child sensibly and not to smother it in clothing because she herself and her mother before her were so smothered. There was no place where tradition was so much out of place as the nursery. Discipline, too, may be inculcated in the Day Nursery. We must remember that since we dwell under artificial conditions it is essential that children should learn to follow well-recognised rules of conduct which have been found useful in communal life.

### Its Psychological Benefit.

Dr. Crichton Miller treated the subject from the point of view of the psychological benefit to children of being at a day nursery. That benefit consisted mainly in the influence of one child upon another. Methods of the day nursery are good for the character of the child, especially for the only child who may be too much the centre of its mother's life. If a mother is not wise and unselfish it is difficult for her to start the child with the capacity for growth of every kind, intellectual and emotional as well as physical. Many only children are moulded not grown. Very few women are big enough to be a wise mother to an only child. It needs far less character to be a wise mother substitute to several children in the Day Nursery.

### Maternity Beds in Hospitals.

"The Available Methods for Securing Healthy Pregnancy and Safe Delivery" were discussed next morning, and Lady Barrett urged that maternity beds should be provided under hospital conditions at a sliding scale of fees for women whose homes were inadequate or unsuitable; and that some provision of specialist's skill, when required, ought to be available for women who were above the hospital class, yet too poor to pay full specialist's fees. In the afternoon Lady Rhondda presided, and aspects of propaganda were debated.

